# Cloning and Expression of Ama r 1, as a Novel Allergen of* Amaranthus retroflexus* Pollen

**DOI:** 10.1155/2016/4092817

**Published:** 2016-01-26

**Authors:** Payam Morakabati, Mohammad-Ali Assarehzadegan, Gholam Reza Khosravi, Bahareh Akbari, Fatemeh Dousti

**Affiliations:** ^1^Department of Immunology, Faculty of Medicine, Ahvaz Jundishapur University of Medical Sciences, Ahvaz 1478736714, Iran; ^2^Department of Immunology, School of Medicine, Iran University of Medical Sciences, Tehran, Iran

## Abstract

Sensitisation to* Amaranthus retroflexus* pollen is very common in tropical and subtropical countries. In this study we aimed to produce a recombinant allergenic Ole e 1-like protein from the pollen of this weed. To predict cross-reactivity of this allergen (Ama r 1) with other members of the Ole e 1-like protein family, the nucleotide sequence homology of the Ama r 1 was investigated. The expression of Ama r 1 in* Escherichia coli* was performed by using a pET-21b(+) vector. The IgE-binding potential of recombinant Ama r 1 (rAma r 1) was evaluated by immunodetection and inhibition assays using 26 patients' sera sensitised to* A. retroflexus* pollen. The coding sequence of the Ama r 1 cDNA indicated an open reading frame of 507 bp encoding for 168 amino acid residues which belonged to the Ole e 1-like protein family. Of the 26 serum samples, 10 (38.46%) had significant specific IgE levels for rAma r 1. Immunodetection and inhibition assays revealed that the purified rAma r 1 might be the same as that in the crude extract. Ama r 1, the second allergen from the* A. retroflexus* pollen, was identified as a member of the family of Ole e 1-like protein.

## 1. Introduction

Pollen from* Amaranthus retroflexus* (redroot pigweed), a well-known species of the Amaranthaceae family which is found throughout the world, is an important trigger of respiratory allergies in different regions with temperate and dry climates, such as Saudi Arabia, Kuwait, India, Iran, the western United States, Australia, and the Mediterranean area [1–4]. Allergy to* A. retroflexus* pollen, one of the most common sources of respiratory allergies among Iranian allergy patients, has also been well defined [1, 5]. This annual weed is abundant in open fields and in farmlands or grasslands. The flowering season of this plant is from around August to October [6].

Immunochemical characterisation of the pollen extract of* A. retroflexus* revealed several components ranging from 10 kDa to 85 kDa [6]. Furthermore, based on the studies of sera of patients with respiratory allergies, the proteins of around 10, 15, 18, 39, 45, and 85 kDa have been reported as IgE-binding proteins from* A. retroflexus* pollen using allergic patients' sera [6, 7]. The first allergen from* A. retroflexus* pollen (Ama r 2) was identified as a member of the profilin family [8]. To the best of our knowledge, despite a high rate of sensitisation to pollens from* Amaranthus* species in different areas of the world [1, 4, 5, 9, 10], few studies about the molecular characterisation of* A. retroflexus* pollen allergens have been conducted to date.

In this study, we introduced Ama r 1, as the second allergen from* A. retroflexus* pollen, which is a member of the Ole e 1-like protein family. The prototypic member of this family is the major olive pollen allergen, Ole e 1 [11]. Several allergens from the Ole e 1-like protein family have been identified previously in other plants, such as* Chenopodium album* (Che a 1) [12],* Salsola kali* (Sal k 5) [13],* Fraxinus excelsior* (Fra e 1) [14],* Ligustrum vulgare* (Lig v 1) [15], and* Syringa vulgaris* (Syr v 1) [16]. In the present study, we aimed to produce Ama r 1 in* Escherichia coli* and then determined the homology of its protein sequence that was determined by comparing it with the most common allergenic Ole e 1-like proteins.

## 2. Materials and Methods

### 2.1. Protein Extraction from* A. retroflexus* Pollen

Flowers of* A. retroflexus* were accumulated from August to October in Ahvaz city, in the southwest of Iran. Gathering of pollen materials and handling were performed by trained pollen collectors. Floral parts other than pollen were separated using the sieves with different sizes (100, 200, and 300 meshes) successively [17].

The final fine powder was subjected to a purity check for pollen content using a microscope. Pollen materials with more than 96% pollen and less than 4% of the other parts of the same plant were gathered for protein extraction. Pollens were defatted using repeated changes of diethyl ether. For protein extraction, two grams of pollen was mixed with 12 mL phosphate-buffered saline (PBS) 0.01 M (pH 7.4) by continuous stirring for 16 h at 4°C. The supernatant was separated by centrifugation at 13,000 ×g for 20 min and filtered and the supernatant collected [18].

The protein content of the extract was evaluated using Bradford's method [19]. Finally, the extract was freeze-dried and stored at −20°C for later use in the present study.

### 2.2. Patients' Sera and Skin Prick Test (SPT)

In this study, we used sera from 26 patients from Ahvaz city, southwest Iran. The patients were 12 men and 14 women (mean age, 29.88 ± 6.88 years; age range, 20–41 years) with respiratory allergies and seasonal rhinitis who had positive skin prick test (SPT) results for* A. retroflexus* pollen extract. Eight subjects without allergies who presented with negative SPTs and no specific IgE to the* A. retroflexus* pollen extract were assigned as negative controls. All patients and control subjects gave us their written informed consent to participate. Serum samples of the subjects were prepared and then immediately stored at −20°C until used.

### 2.3. Total IgE and Specific Indirect Enzyme-Linked Immunosorbent Assays (ELISA)

Total serum IgE levels were evaluated in serum samples in duplicate using a commercially available ELISA kit according to the manufacturer's instructions (Euroimmun, Lübeck, Germany). To evaluate the levels of specific IgE to* A. retroflexus* pollen proteins in patients with respiratory allergies, an indirect ELISA was devised. In brief, wells of an ELISA microplate (Nunc A/S, Roskilde, Denmark) were coated with 100 *μ*L/well of* A. retroflexus* pollen extract [10 *μ*g/well in coating buffer (15 mM Na_2_CO_3_, 35 mM NaHCO_3_, pH 9.6)] at 4°C for overnight. After blocking with a solution composed of 150 *μ*L of phosphate buffered saline (PBS) and 2% bovine serum albumin (BSA) solution for 1 hour at 37°C, the plates were incubated with 100 *μ*L of patients' sera for 3 hours at room temperature. Each well was then incubated for 2 hours at room temperature with a 1 : 500 dilution of biotinylated goat anti-human IgE antibody (Nordic-MUbio, Susteren, Netherlands) in 1% PBS. The wells were then washed five times with PBS containing 0.05% Tween-20 (T-PBS) to eliminate unbound anti-IgE. Thereafter, 100 *μ*L of horseradish peroxidase- (HRP-) conjugated streptavidin (Bio-Rad Laboratories, Hercules, CA, USA) was placed in each well, and each well was then incubated for 1 hour at room temperature. After five washes with T-PBS, each well received 100 *μ*L of tetramethylbenzidine substrate solution (Sigma-Aldrich, St. Louis, MO, USA) that was placed in each well, and the plate was incubated at room temperature for 20 min before the reaction was stopped by addition of 100 *μ*L of 2 M HCl. Subsequently, the absorbance in each well was measured at 450 nm using an ELISA plate reader. All results were expressed as optical density (OD) units. An OD value four times higher than the average values of three determinations of a pooled sera from negative controls (i.e., >0.15 OD units) was considered to be positive.

### 2.4. Subcloning of Ama r 1 Allergen cDNA and DNA Sequencing

Total RNA was extracted from 100 mg of* A. retroflexus* pollen by using the Chomczynski and Sacchi method [20]. The first strand of cDNA was synthesised using the RevertAid*™* First Strand cDNA Synthesis Kit (Thermo Scientific, Waltham, MA, USA) according to the manufacturer's instructions. The primers used for cDNA amplification were designed according to the known nucleotide sequence for reported allergens from the Ole e 1-like protein family that have a high degree of amino acid sequence identity [12–14, 21–23]. These primers include the sense 5′-ATGGGGAAGTGTCAAGCTGT-3′ and the antisense 5′-TTAATTAGCTTTAACATCATAAAGATCC-3′. The amplified fragment was ligated into a PTZ57R/T TA cloning vector using the InsTAclone*™* PCR Cloning Kit (Thermo Scientific, Waltham, MA, USA), according to the manufacturer's instructions.* E. coli* TOP10 cells (Invitrogen, Carlsbad, CA, USA) were transformed with the ligation products using the manufacturer's protocol. A recombinant plasmid was selected by white/blue screening and then purified from the gel using a plasmid extraction kit (GeNet Bio, Chungnam, Korea). DNA sequence analysis was performed using the dideoxy method at the Bioneer Inc. (Daejeon, Korea).

The obtained sequence was submitted to the GenBank database of the National Center for Biotechnology Information (http://www.ncbi.nlm.nih.gov/) under accession number KR870437.

### 2.5. Construction of Prokaryote Expression Vector and Production of Recombinant Ama r 1 (rAma r 1)

To create a pET-21b(+) expression plasmid (Novagen, Gibbstown, NJ, USA), restriction sites of* Not* I and* Xho* I restriction enzyme sites were introduced at the 5′- and 3′-ends of the Ama r 1 cDNA by the polymerase chain reaction (PCR) method using two specific primers as follows: the sense primer 5′-TCCgcggccgcATGGGGAAGTGTCAAGCTGT-3′ (*Not* I restriction site is in lowercase) and the antisense primer 5′-CCctcgagTTAATTAGCTTTAACATCATAAAGATCC-3′ (*Xho* I restriction site is in lowercase). After PCR amplification, the resulting product was digested with* Not* I and* Xho* I restriction enzymes according to the manufacturer's protocol (Thermo Scientific, Waltham, MA, USA). The purified digested PCR product was ligated into the digested pET-21b(+) plasmid with the same enzymes. Correct constructs were transformed into competent* E. coli* BL21 (DE3) cells (Novagen, Gibbstown, NJ, USA).

A clone of recombinant plasmid pET-21b(+)/Ama r 1 was inoculated into 2 mL of lysogeny broth (LB) medium containing 100 *μ*g/mL of ampicillin and incubated at 37°C. Expression of the recombinant protein was induced by adding isopropyl *β*-d-thiogalactopyranoside (IPTG) to a final concentration of 0.5 mM [18]. Afterwards, the cells were harvested by centrifugation (3,500 ×g for 15 min at 4°C), resuspended in lysis buffer (50 mM Tris-HCl pH 6.8, 15 mM imidazole, 100 mM NaCl, 10% glycerol, and 0.5% Triton X-100), and then disrupted by sonication. Purification of rAma r 1 was performed with Ni-nitrilotriacetic acid (NTA) agarose (Invitrogen, Carlsbad, CA, USA) from the soluble phase of lysate according to the manufacturer's instructions.

### 2.6. Determination of Specific IgE Levels to rAma r 1

In order to assess the serum IgE levels to the purified rAma r 1, an indirect ELISA was developed as described above, except that the wells of the ELISA microplate were coated with 100 *μ*L/well of the purified rAma r 1 at a concentration of 2 *μ*g/mL in coating buffer (15 mM Na_2_CO_3_ and 35 mM NaHCO_3_, pH 9.6) overnight at 4°C. The results were expressed in OD units. Based on the mean value of two normal sera, OD_450_ greater than three times the median values of negatives controls was considered to be positive.

### 2.7. ELISA Inhibition Assays for rAma r 1

ELISA inhibition was performed as described above, except for a pooled serum (1 : 2 vol/vol) from patients allergic to* A. retroflexus* (patients 1, 2, 4, 6, and 8), which was preincubated for overnight at 4°C with either 1000, 100, 10, 1, 0.1, or 0.01 *μ*g of rAma r 1 as an inhibitor or with BSA as a negative control. The inhibition percentage was calculated using the following relationship:

(1) $$\begin{array}{c} \%\;\;Inhibition=\left(\;\frac{OD\;\;of\;\;sample\;\;without\;\;inhibitor-OD\;\;of\;\;sample\;\;with\;\;inhibitor}{OD\;\;of\;\;sample\;\;without\;\;inhibitor}\;\right)\times 100. \end{array}$$

### 2.8. IgE-Immunoblotting and IgE-Immunoblotting Inhibition for rAma r 1

Proteins from* A. retroflexus* pollen extract and* E. coli* lysate and purified rAma r 1 were analysed by sodium dodecylsulphate polyacrylamide gel electrophoresis (SDS-PAGE) using 12.5% acrylamide separation gels and under reducing conditions according to the method of Laemmli [24]. The molecular masses of protein bands were estimated with Image Lab analysis software (Bio-Rad Laboratories, Hercules, CA, USA) and compared with protein markers of known molecular weights (Amersham low molecular weight calibration kit for SDS electrophoresis; GE Healthcare, Little Chalfont, UK). Separated protein bands from the electrophoresis of* A. retroflexus* pollen were electrotransferred to polyvinylidene difluoride (PVDF) membranes (GE Healthcare, Little Chalfont, UK), as described elsewhere [18]. In brief, after blocking and washing, membranes were incubated with a serum pool or individual sera from patients with* A. retroflexus* allergy or with control sera (1 : 5 dilutions) for 3 hours. Biotinylated goat anti-human IgE (Nordic-MUbio, Susteren, Netherlands) (1 : 1000 vol/vol in PBS) was added to the blotted membrane strips and incubated for 2 hours at room temperature. The unbound antibodies were removed from blots by washing with T-PBS and incubated at 1 : 10,000 vol/vol in T-PBS-HRP-linked streptavidin (Sigma-Aldrich, St. Louis, Mo, USA) for 1 hour at room temperature. After several washes with T-PBS, strips were incubated using the SuperSignal West Pico Chemiluminescent Substrate Kit (Thermo Scientific, Waltham, MA, USA) for 5 minutes, and proteins were then visualised by chemiluminescence using the ChemiDoc XRS+ System (Bio-Rad Laboratories, Hercules, CA, USA).

To study cross-inhibition among natural and recombinant Ama r 1, a mixture of 100 *μ*L of pooled serum (1 : 5 vol/vol) was incubated with natural* A. retroflexus* pollen extract (65 *μ*g/mL, as an inhibitor), rAma r 1 (10 *μ*g/mL, as an inhibitor), or BSA (as a negative control) overnight at 4°C with shaking. Preincubated sera were used to assess the reactivity of a PVDF membrane blotted with natural* A. retroflexus* pollen extract and rAma r 1.

## 3. Results

### 3.1. Measurement of Total and Specific IgE

The mean total IgE serum in the subjects was determined as 256.33 IU/mL. In patients reactive to Ama r 1, the mean of total IgE was 183.80 IU/mL (Table 1). Sera from 26 allergic patients were assessed for specific IgE binding to proteins from* A. retroflexus* pollen extract. All of these patients had significantly elevated specific IgE levels to the extract of* A. retroflexus* pollen (mean OD_450_ = 1.47 ± 0.50; range, 0.79–2.21). The mean OD_450_ for specific IgE in patients reactive to rAma r 1 was 0.95 ± 0.16 (range, 0.78–1.23) (Table 1).

### 3.2. Nucleotide and Protein Sequence Analysis of Ama r 1

**Figure 1:**
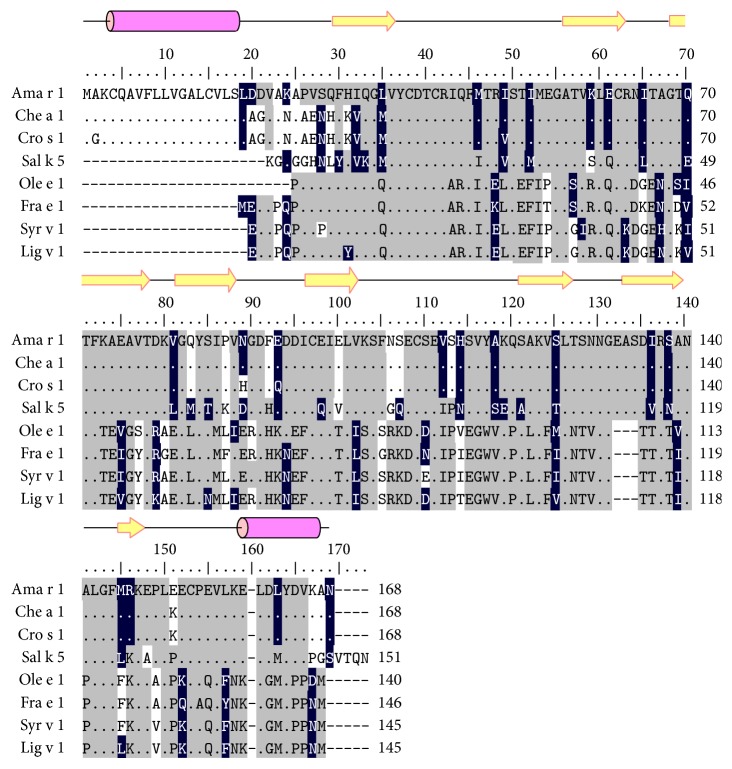
Comparison of the* A. retroflexus* Ole e 1-like protein (Ama r 1) amino acid sequence with allergenic Ole e 1-like protein from other plants.* Chenopodium album* (Che a 1, G8LGR0.1),* Crocus sativus* (Cro s 1, XP004143635.1),* Salsola kali* (Sal k 5, ADK22842.1),* Olea europaea* (Ole e 1, P19963.2),* Fraxinus excelsior* (Fra e 1, AAQ83588.1),* Syringa vulgaris* (Syr v 1, S43243), and* Ligustrum vulgare* (Lig v 1, O82015.2). The amino acid sequence identity and the similarity of Ama r 1 (KR870437) to other members of the Ole e 1-like family are shown in Table 2. The top line indicates the location of secondary structures that are created by PSIPRED protein sequence analysis (http://bioinf.cs.ucl.ac.uk/psipred/). The cylinder, arrows, and black line correspond to alpha-helices, beta-strands, and coil structure, respectively.

The sequence analysis of Ama r 1 indicated an open reading frame of 507 bp coding for 168 amino acid residues with a predicted molecular mass of 18.379 kDa and a calculated isoelectric point (pI) of 4.70. We compared the deduced amino acid sequence of Ama r 1 with other allergenic plant-derived Ole e 1-like proteins in the protein database (Figure 1), and we detected a high level of sequence identity (93%) that was detected between Ama r 1 and Che a1 (Table 2).

### 3.3. SDS-PAGE and IgE-Binding Components of* A. retroflexus* Pollen Extract

**Figure 2:**
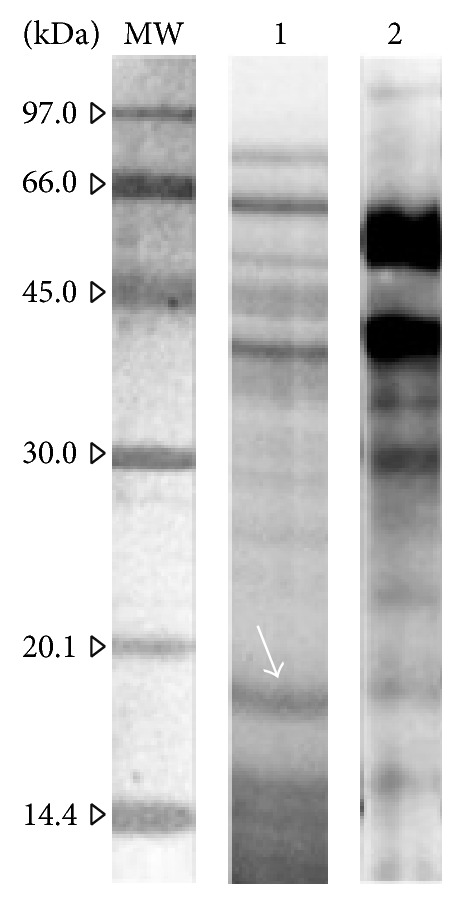
SDS-PAGE and immunoreactivity of* A. retroflexus* pollen extract.* Lane MW*: molecular weight marker (GE Healthcare, Little Chalfont, UK);* lane 1*: Coomassie Brilliant Blue-stained SDS-PAGE of the crude extract of* A. retroflexus* pollen (12.5% acrylamide gel); and* lane 2*: immunoblotting of* A. retroflexus* pollen extract. The strip was first blotted with* A. retroflexus* pollen extract and then incubated with pooled sera of patients allergic to* A. retroflexus* (patients 1, 2, 4, 6, and 8) and detected for IgE reactive protein bands. Natural Ama r 1 is indicated by* white arrow*.

The reducing SDS-PAGE separation of the pollen extract showed several resolved protein bands in the* A. retroflexus* pollen extract with molecular weights ranging from approximately 10 to 85 kDa (Figure 2). IgE-binding reactivity of the separated protein bands from the electrophoresis of the* A. retroflexus* pollen extract was assessed by conducting immunoblotting experiments. The results revealed that several IgE-reactive bands range from about 15 to 85 kDa.

### 3.4. Expression and Purification of Ama r 1 Protein

**Figure 3:**
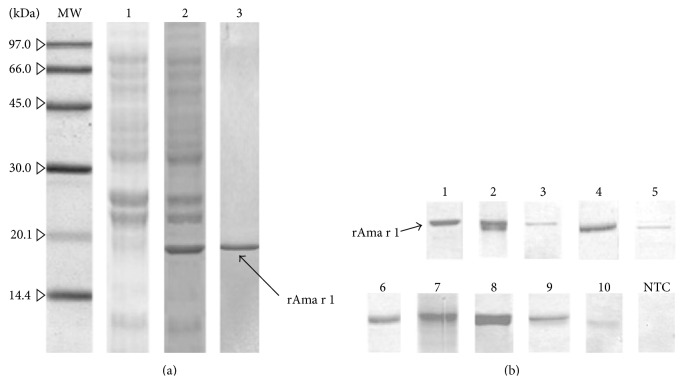
SDS-PAGE and immunoreactivity of recombinant Ama r 1 (rAma r 1). (a)* Lane MW*: molecular weight marker (GE Healthcare, Little Chalfont, UK);* lane 1*: Coomassie Brilliant Blue-stained SDS-PAGE of soluble fraction of cell culture (IPTG-induced pET-21b(+) without insert);* lane 2*: rAma r 1 (IPTG-induced pET-21b(+)/Aca f 1) in soluble fraction; and* lane 3*: purified rAma r 1 (as an approximately 19 kDa recombinant protein) with Ni-NTA affinity chromatography on 12.5% acrylamide gel. (b) IgE immunoblot of purified rAma r 1 using sera of patients with respiratory allergies.* Lanes 1–10*: probed with sera from patients with positive for rAma r 1;* lane NTC*: negative control.

A pET-21b (+)/Ama r 1 clone was constructed and confirmed by digestion with* Not* I and* Xho* I restriction enzymes. This recombinant plasmid was expressed in* E. coli* strain BL21 (DE3) pLysS as a fusion protein with a His_6_-tag in the C-terminus. rAma r 1 was present in a soluble form in the supernatant, where it was further purified by Ni^2+^ affinity chromatography to yield purified protein. The purified rAma r 1 was quantified by using Bradford's protein assay, which showed that approximately 17 mg of recombinant protein had been purified from 1 L of the bacterial expression medium. SDS-PAGE revealed that the apparent molecular weight of the fusion protein was about 19 kDa (Figure 3). The allergenic Ole e 1-like protein from* A. retroflexus* pollen, as a new allergen, was designated Ama r 1 by the WHO/International Union of Immunological Societies (IUIS) Allergen Nomenclature Subcommittee (http://www.allergen.org/).

### 3.5. Specific IgE ELISA of rAma r 1

The levels of specific IgE to the purified rAma r 1 were determined using 26 individual patients' sera. Of the 26 patients, 10 (38.46%) had significant specific IgE levels to rAma r 1 (Table 1). Serum samples from the patients allergic to* A. retroflexus* pollen were further tested for IgE reactivity to rAma r 1 by immunoblotting assays. The results showed that the recombinant form of Ama r 1 was reactive with 10 individuals' sera. These results were consistent with those obtained by specific IgE ELISA (Table 1).

### 3.6. *In Vitro* Inhibition Assays

**Figure 4:**
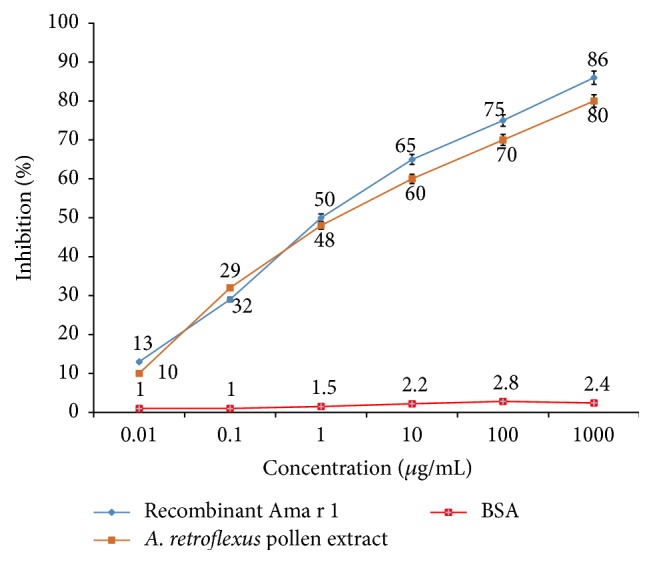
ELISA inhibition with* A. retroflexus* pollen extract and rAca f 1. Inhibition of IgE binding to rAca f 1 by ELISA using* A. retroflexus* pollen extract and rAma r 1. Control experiments were performed with BSA.

ELISA inhibition experiments were performed to evaluate the IgE-binding capacity of the purified rAma r 1 compared with its natural counterpart in* A. retroflexus* pollen extract. The ELISA inhibition results revealed a dose-dependent inhibition of the IgE directed towards rAma r 1 in patients' sera positive to* A. retroflexus*. Preincubation of pooled sera with 1 mg/mL of rAma r 1 and* A. retroflexus* pollen extract showed significant inhibition (86% and 80%, resp.) of IgE binding to rAma r 1 in microplate wells (Figure 4).

**Figure 5:**
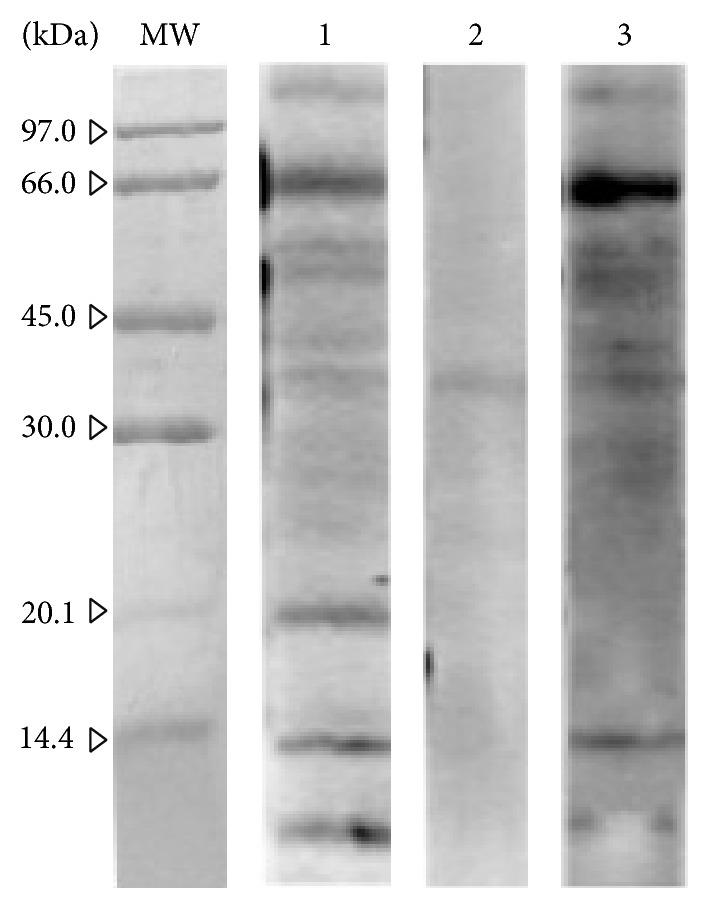
Immunoblotting inhibition assays.* Lane MW*: molecular weight marker (GE Healthcare, UK);* lane 1*:* A. retroflexus* protein strip incubated with pooled sera without inhibitor (negative control);* lane 2*:* A. retroflexus* protein strip incubated with pooled sera containing 65 *μ*g of* A. retroflexus* pollen extract as an inhibitor (positive control); and* lane 3*:* A. retroflexus* protein strip incubated with pooled sera containing 10 *μ*g purified rAma r 1, as an inhibitor.

Immunoblot inhibition assays indicated that preincubation of serum samples with rAma r 1 almost completely inhibited the IgE binding to a protein band with an apparent molecular weight of 19 kDa (Figure 5, line 3). Altogether,* in vitro* inhibition assays showed a similar IgE reactivity for rAma r 1 and its natural counterpart in* A. retroflexus* pollen extract. In addition, the results indicated that preincubation of serum samples with native crude extract of* A. retroflexus* pollen completely inhibited the IgE binding to natural Ama r 1 counterparts in* A. retroflexus* pollen extract and other reactive proteins (Figure 5, line 2). However, preincubation of the pooled sera with BSA did not affect the IgE reactivity to rAma r 1 (Figure 5, line 1).

## 4. Discussion

*A. retroflexus* is a weed broadly distributed across wastelands and farms in various climates, and it produces such a large quantity of pollen that it has become one of the most allergenic weeds in different countries throughout the world [1–5, 10]. In this study, the cloning and production of the second allergen of the* A. retroflexus* pollen is reported. This allergen was shown to be a member of Ole e 1-like protein family, and, in accordance with the IUIS Allergen Nomenclature Subcommittee, was designated as Ama r 1. Several allergens from this family, such as Sal k 5, Che a 1, Cro s 1, Pla l 1, Syr v 1, Lig v 1, and Fra e 1, have been recognised in previous studies [12–14, 21–23].

The open reading frame of Ama r 1 contained a sequence encoding an 18.37 kDa protein related to the molecular specifications of a known plant Ole e 1-like protein family [13, 14, 21]. Until now, several members of Ole e 1-like protein allergens from different plant sources have been reported with various molecular weights, such as 17.08–17.62 kDa in two members of the Amaranthaceae family (Che a 1, Sal k 5), 20 kDa in* C. sativus* pollen (Cro s 1), and 17–20 kDa (glycosylated and nonglycosylated) in* Plantago lanceolata* (Pla l 1) [13, 22, 25]. These relative disparities in molecular weight may be due to differences in some amino acid residues, levels of glycosylation, or molecular weight measurement methods MWs methods. Ama r 1, like Che a 1, Cro s 1, and Sal k 5, has a conserved sequence for potential N-linked glycosylation in the same position of the polypeptide chain (Asn-Ile/Leu-Thr-Ala), which is actually engaged by a glycan in these proteins.

Immunoblotting assays of* A. retroflexus* pollen extract using pooled sera from the patients also revealed an IgE-binding protein band with an estimated molecular weight of 19 kDa (Figure 1). The IgE-binding capability of the purified rAma r 1 to sera from patients with* A. retroflexus* allergies was evaluated by specific ELISA and immunoblotting assays to confirm that rAma r 1 was correctly folded and bound to IgE as the natural counterpart in* A. retroflexus* extract. The purified rAma r 1 was recognised in 10 patients allergic to the* A. retroflexus* pollen extract (10/26, 38.4%).

The results of immunoblotting assays for natural Ama r 1 with a molecular weight of 19 kDa were consistent with those obtained for rAma r 1. A nearly complete inhibition of IgE-binding to natural Ama r 1 was also obtained after preincubation of pooled sera with purified rAma r 1. It seems that rAma r 1 is composed of IgE epitopes similar to those of its natural counterpart.

Cross-reactivity between* A. retroflexus* pollen components and other allergenic members of the Amaranthaceae family (*S. kali*,* C. album*, and* Kochia scoparia*) and some unrelated allergenic plants such as* Acacia farnesiana* and* Prosopis juliflora* has been described previously [6, 7, 17]. The present study was conducted to detect the amino acid sequence homology of Ole e 1-like proteins from allergenic regional plants. The results of amino acid sequence identity analysis indicated that Ama r 1 protein has a great degree of identity with the selected allergenic Ole e 1-like protein family from the most common allergenic regional plants, particularly* C. album* (Che a 1),* C. sativus* (Cro s 1), and* S. kali* (Sal k 5) (93%, 91%, and 70%, resp.). Identification of the Ama r 1 sequence will warrant further studies on the basis of* in vitro* assays to investigate the molecular basis of cross-reactivity among these important pollen allergens.

## 5. Conclusion

In conclusion, in this study, we investigated a new allergen from* A. retroflexus* pollen, Ama r 1, with a detectably specific IgE in 38.4% of patients allergic to* A. retroflexus* pollen. Ama r 1 was identified as a member of the Ole e 1-like protein family. In addition, the results demonstrate that rAma r 1 expressed in* E. coli* has immunoreactivity similar to that of the natural form of the allergen. Analysis of the amino acid sequences of Ama r 1 and several allergenic members of the Ole e 1-like protein family from other plants also indicated that cross-reactivity between plants belongs to unrelated families, which may be predicted by the degree of amino acid sequence identity of potential conformational epitopes.

Concerning the more prevalent of sensitisation to* A. retroflexus* pollen and the abundance of it in different countries throughout the world, identification and production of the recombinant forms of common allergens of this pollen may lead to the exploration of new guidelines for diagnostic, therapeutic, and preventive purposes.

## Figures and Tables

**Table 1:** Clinical attribute, skin prick test responses, and specific IgE values of patients reactive to recombinant Ama r 1.

Patients number	Age (years)/gender^1^	Symptoms^2^	Total IgE (IU/mL)	*A. retroflexus* pollen extract		rAma r 1-specific IgE
				Skin prick test^3^	Specific IgE^4^	
1	38/M	A, R	152	8	1.80	0.95
2	32/F	A, R, L	185	12	2.10	1.23
3	21/F	A, R	162	8	0.98	0.80
4	38/F	A, R, L	224	12	1.89	1.10
5	23/M	A, R, L	132	10	1.10	0.84
6	29/F	A, R, L	166	12	1.95	0.98
7	41/M	A, R	175	9	0.92	0.81
8	32/F	A, R, L	305	15	2.21	1.15
9	22/M	A, R	159	11	1.12	0.87
10	35/F	A, L	178	10	0.97	0.78

^1^M: male; F: female.

^2^A: allergic rhinitis; L: lung symptoms (breathlessness, tight chest, cough, and wheeze); R: rhinoconjunctivitis.

^3^The mean wheal areas are displayed in mm^2^. Histamine diphosphate (10 mg/mL)—positive control; Glycerin—negative control.

^4^Determined in specific ELISA as OD (optical density) at 450 nm.

**Table 2:** Percentage of similarity and identity between Ama r 1 and selected allergenic Ole e 1-like proteins.

Allergens^*∗*^	GenBank accession number	Ama r 1	
		% Similarity	% Identity
Che a 1	G8LGR0.1	95	93
Cro s 1	AAX93750.1	95	91
Sal k 5	ADK22842.1	88	70
Ole e 1	ABP58635.1	61	43
Fra e 1	AAQ83588.1	62	42
Lig v 1	O82015.2	61	40
Syr v 1	S43243	60	42

^*∗*^Che a 1 (*C. album*); Cro s 1 (*C. sativus*); Sal k 5 (*S. kali*); Ole e 1 (*O. europaea*); Fra e 1 (*F. excelsior*); Syr v 1 (*S. vulgaris*); and Lig v 1 (*L. vulgare*).

## References

[1] Assarehzadegan M.-A., Shakurnia A., Amini A. (2013). The most common aeroallergens in a tropical region in Southwestern Iran. *World Allergy Organization Journal*.

[2] Ghosh D., Chakraborty P., Gupta J. (2012). Associations between pollen counts, pollutants, and asthma-related hospital admissions in a high-density indian metropolis. *Journal of Asthma*.

[3] Min K., Yoshida M., Miike R., Tam E. (2014). Aeroallergen sensitivity in Hawaii: association with asthma and increased prevalence of sensitivity to indoor allergens Since 1996. *Hawaii Journal of Medicine & Public Health*.

[4] Villalba M., Barderas R., Mas S., Colás C., Batanero E., Rodríguez R. (2014). Amaranthaceae pollens: review of an emerging allergy in the mediterranean area. *Journal of Investigational Allergology and Clinical Immunology*.

[5] Assarehzadegan M.-A., Shakurnia A. H., Amini A. (2013). Sensitization to common aeroallergens among asthmatic patients in a tropical region affected by dust storm. *Journal of Medical Sciences*.

[6] Tehrani M., Sankian M., Assarehzadegan M. A., Falak R., Jabbari F., Varasteh A. (2010). Immunochemical characterization of *Amaranthus retroflexus* pollen extract: extensive cross-reactive allergenic components among the four species of Amaranthaceae/Chenopodiaceae. *Iranian Journal of Allergy, Asthma and Immunology*.

[7] Wurtzen P. A., Nelson H. S., Lowenstein H., Ipsen H. (1995). Characterization of chenopodiales (*Amaranthus retroflexus, Chenopodium album, Kochia scoparia, Salsola pestifer*) pollen allergens. *Allergy*.

[8] Tehrani M., Sankian M., Assarehzadegan M. A. (2011). Identification of a new allergen from *Amaranthus retroflexus* pollen, Ama r 2. *Allergology International*.

[9] Hasnain S. M., Al-Frayh A. R., Subiza J. L. (2012). Sensitization to indigenous pollen and molds and other outdoor and indoor allergens in allergic patients from Saudi Arabia, United Arab Emirates, and Sudan. *World Allergy Organization Journal*.

[10] Singh A. B., Dahiya P. (2002). Antigenic and allergenic properties of *Amaranthus spinosus* pollen—a commonly growing weed in India. *Annals of Agriculture and Environmental Medicine*.

[11] de Dios Alché J., M'rani-Alaoui M., Castro A. J., Rodríguez-García M. I. (2004). Ole e 1, the major allergen from olive (*Olea europaea* L.) pollen, increases its expression and is released to the culture medium during in vitro germination. *Plant and Cell Physiology*.

[12] Barderas R., Villalba M., Lombardero M., Rodríguez R. (2002). Identification and characterization of Che a 1 allergen from *Chenopodium album* pollen. *International Archives of Allergy and Immunology*.

[13] Castro L., Mas S., Barderas R. (2014). Sal k 5, a member of the widespread ole e 1-like protein family, is a new allergen of russian thistle (*Salsola kali*) pollen. *International Archives of Allergy and Immunology*.

[14] Barderas R., Purohit A., Rodríguez R., Pauli G., Villalba M. (2006). Isolation of the main allergen Fra e 1 from ash (*Fraxinus excelsior*) pollen: comparison of the natural and recombinant forms. *Annals of Allergy, Asthma & Immunology*.

[15] Batanero E., Gonzalez De La Peña M. A., Villalba M., Monsalve R. I., Martin-Esteban M., Rodriguez R. (1996). Isolation, cDNA cloning and expression of Lig v 1, the major allergen from privet pollen. *Clinical and Experimental Allergy*.

[16] González E., Villalba M., Rodríguez R. (2001). Immunological and molecular characterization of the major allergens from lilac and privet pollens overproduced in *Pichia pastoris*. *Clinical and Experimental Allergy*.

[17] Shamsbiranvand M. H., Khodadadi A., Assarehzadegan M.-A., Borci S. H., Amini A. (2014). Immunochemical characterization of acacia pollen allergens and evaluation of cross-reactivity pattern with the common allergenic pollens. *Journal of Allergy*.

[18] Zarinhadideh F., Amini A., Assarehzadegan M. A., Borsi S. H., Sepahi N., Ali-Sadeghi H. (2015). Immunochemical and molecular characterization of allergenic profilin (Koc s 2) from *Kochia scoparia* pollen. *Journal of the Korean Society for Applied Biological Chemistry*.

[19] Bradford M. M. (1976). A rapid and sensitive method for the quantitation of microgram quantities of protein utilizing the principle of protein-dye binding. *Analytical Biochemistry*.

[20] Chomczynski P., Sacchi N. (1987). Single-step method of RNA isolation by acid guanidinium thiocyanate-phenol-chloroform extraction. *Analytical Biochemistry*.

[21] Asturias J. A., Arilla M. C., Gómez-Bayón N., Martínez J., Martínez A., Palacios R. (1997). Cloning and expression of the panallergen profilin and the major allergen (Ole e 1) from olive tree pollen. *Journal of Allergy and Clinical Immunology*.

[22] Calabozo B. N., Díaz-Perales A., Salcedo G., Barber D., Polo F. (2003). Cloning and expression of biologically active *Plantago lanceolata* pollen allergen Pla l 1 in the yeast *Pichia pastoris*. *Biochemical Journal*.

[23] Varasteh A.-R., Moghadam M., Vahedi F., Kermani T., Sankian M. (2009). Cloning and expression of the allergen Cro s 2 profilin from saffron (*Crocus sativus*). *Allergology International*.

[24] Laemmli U. K. (1970). Cleavage of structural proteins during the assembly of the head of bacteriophage T4. *Nature*.

[25] Barderas R., Villalba M., Rodríguez R. (2004). Che a 1: recombinant expression, purification and correspondence to the natural form. *International Archives of Allergy and Immunology*.

